# Failure Mechanism and Control Countermeasures for Argillaceous Surrounding Rock of Horsehead Roadway under High Stress

**DOI:** 10.3390/ma16114180

**Published:** 2023-06-04

**Authors:** Deyu Qian, Qi Cui, Hexi Jiao, Guanghui Zhu, Zhiyi Zhang, Linyou Jiang, Qingbin Meng, Jiale Liu, Xing Gao, Fujia Xing

**Affiliations:** 1 School of Mines, China University of Mining and Technology, Xuzhou 221116, China; ts20020092p21@cumt.edu.cn (Q.C.); ts21020018a31tm@cumt.edu.cn (H.J.);; 2 China Coal Huajin Group, Hanzui Coal Industry Co., Ltd., Linfen 042100, China; 3 China Coal Huajin Group, Jincheng Energy Co., Ltd., Jincheng 048200, China; 15234669787@163.com; 4 School of Geology and Mining Engineering, Xinjiang University, Urumchi 830046, China; 5 State Key Laboratory for Geomechanics and Deep Underground Engineering, China University of Mining and Technology, Xuzhou 221116, China

**Keywords:** horsehead roadway, argillaceous surrounding rock, deformation and failure, prestressed full-length anchorage, innovative anchor-grouting device, reverse arch

## Abstract

The argillaceous surrounding rock of a horsehead roadway under high stress conditions is prone to deformation and failure, and the control of its long-term stability is difficult. Based on the engineering practices that control the argillaceous surrounding rock of a horsehead roadway in the return air shaft in the Libi Coal Mine in Shanxi Province, field measurements, laboratory experimentation, numerical simulation, and industrial tests are used to analyze the main influencing factors and mechanism of the deformation and failure of the surrounding rock of the horsehead roadway. We propose principles and countermeasures to control the stability of the horsehead roadway. The main factors of the surrounding rock failure of the horsehead roadway include the poor lithology of argillaceous surrounding rocks, horizontal tectonic stress, the superimposed influence of additional stress from the shaft and construction disturbance, the small thickness of the anchorage layer in the roof, and the insufficient depth of floor reinforcement. The results show that the shaft’s presence increases the horizontal stress peak and stress concentration range in the roof, and the plastic zone range. The stress concentration and plastic zones and deformations of the surrounding rock increase significantly with the increase in horizontal tectonic stress. The control principles for the argillaceous surrounding rock of the horsehead roadway include increasing the thickness of the anchorage ring, the floor reinforcement exceeding the minimum depth, and reinforced support in key positions. The key control countermeasures include an innovative prestressed full-length anchorage for the mudstone roof, active and passive reinforcement technology with cables, and a reverse arch for floor reinforcement. The field measurements show that the control of the surrounding rock using the prestressed full-length anchorage of the innovative anchor-grouting device is remarkable.

## 1. Introduction

As the connecting channel between the shaft, the pit bottom, and the main entry, the horsehead roadway is called the “throat” of mine production. It is the lifeline of mine safety production, and its stability is crucial [1]. However, the argillaceous surrounding rock of a horsehead roadway can deform and be destroyed easily due to its long service life, large cross-section, the influence of additional stress from the shaft, and the high stress concentration of the surrounding rock. There is a problem of long-term stability control, which considerably restricts safety processes during the construction and the operation of the mine [2,3].

Several studies have been conducted on the deformation and damage mechanisms and control techniques for large-section underground openings such as the horsehead roadway [4,5,6,7,8]. Song et al. [9] studied the effect of the magnitude and direction of the principal stress on the deformation law of the roof, floor, and two sides of a horsehead roadway in the context of its weakly cemented surrounding rock. Liu et al. [10] used a stress monitoring system to conduct comprehensive monitoring and obtained the evolution law of the deformation and fracture of the surrounding rock of a deep super-large cross-section cavern group. Tan et al. [11,12] obtained the basis of the instability of a large-section chamber group and the evolution law of the stress of the surrounding rock by establishing a mechanical model. Meng et al. [13] analyzed the evolution law of the displacement field and plastic zone of the surrounding rock of a skip-loading chamber under the conditions of excavation and support and revealed the evolution law of bolt axial force, surrounding rock pressure, and secondary lining steel force with time. Wang et al. [14] systematically studied the effects of different factors on the deformation control of surrounding rock, such as the relative position and excavation sequence of the chamber, the bolt-grouting support, and the reinforcement support of the key parts, proposing a method for optimizing the design of deep large-section chambers.

The above research results have solved the technical problems of large-section chamber support to a certain extent; however, after the excavation of the horsehead roadway in high-stress mudstone, the fracture zone is large, the deformation is intense, the roof is prone to delamination, the bolt–shotcrete support is prone to failure, and there is a potential risk of the large-scale roof falling, which is not conducive to the control of the surrounding rock of a large-section chamber [15,16,17]. Based on the research background of the horsehead roadway project surrounded by argillaceous rock in the return air shaft of the Libi Coal Mine in Shanxi Province, this paper analyzes the deformation and failure characteristics and main influencing factors of the horsehead roadway surrounded by argillaceous rock under high stress, studies the influence law of additional stress and horizontal tectonic stress on the stability of the horsehead roadway surrounding rock, and proposes control principles and countermeasures for the stability of the surrounding rock. The research results have important theoretical value and engineering significance for the control of the stability of large-section chambers, such as horsehead roadways under high stress.

## 2. Engineering Geological Profiles of the Horsehead Roadway

### 2.1. Engineering Geology of the Horsehead Roadway

**Figure 1:**
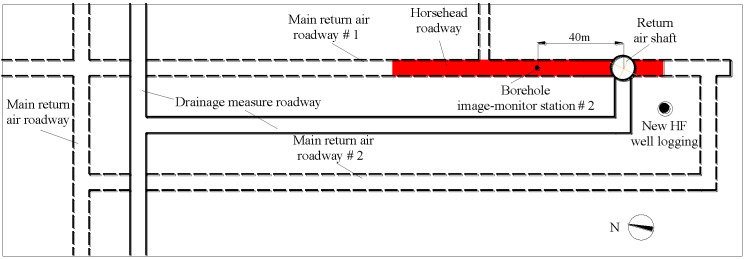
Layout of the horsehead roadway in the return air shaft.

The Libi Coal Mine is located in Longgang Town, Qinshui County, Jincheng City. The buried depth of the horsehead roadway of the return air shaft is about 537.37 m. The roof of the horsehead roadway is mudstone. The surrounding rock of the sidewalls consists of limestone, mudstone, and sandy mudstone. The lithology in the floor is sandy mudstone and fine sandstone. The layout plan of the horsehead roadway is shown in Figure 1. The red section in the figure is the roof and floor deformation and the reinforcement area.

### 2.2. The Original Support Scheme for the Horsehead Roadway

**Figure 2:**
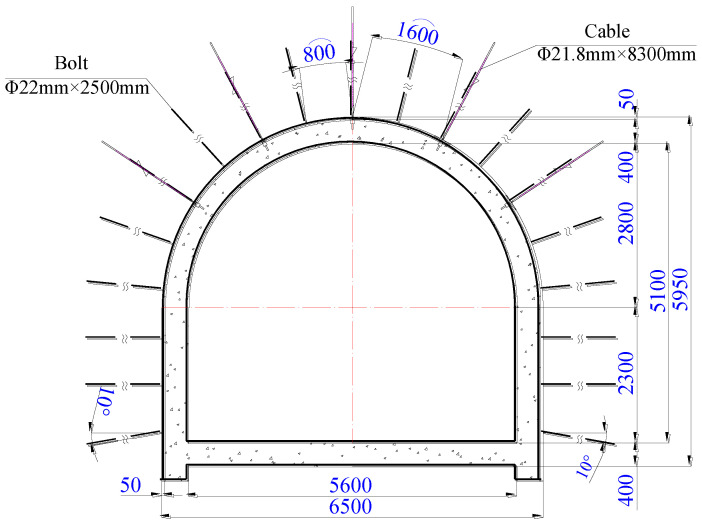
Support section.

The size of the excavation section of horsehead roadway was 6500 mm in width and 5950 mm in height. The excavation of the roadway was carried out in full section. The cycle footage of the excavation and support is 1600 mm. The bolts and cables were carried out after excavation. The bolts and cables were 800 mm and 1600 mm away from the tunneling face, respectively. The original support scheme from the coal mine design institute was an anchor net spray support and reinforced concrete lining. The support parameters are shown in Figure 2. There were thirteen thread steel bolts in the roof and three on both sidewalls, each 22 mm in diameter and 2500 mm in length. The interval and spacing of the bolts were both 800 mm. There were five anchor cables in the roof, each 21.8 mm in diameter and 8300 mm in length. The interval and spacing of the bolts were both 1600 mm.

The thickness of the reinforced concrete lining was 400 mm. The concrete strength was C50. The size of the net section was 5600 mm in width and 5100 mm in height.

### 2.3. Deformation and Failure Characteristics of the Surrounding Rock of the Horsehead Roadway

**Figure 3:**
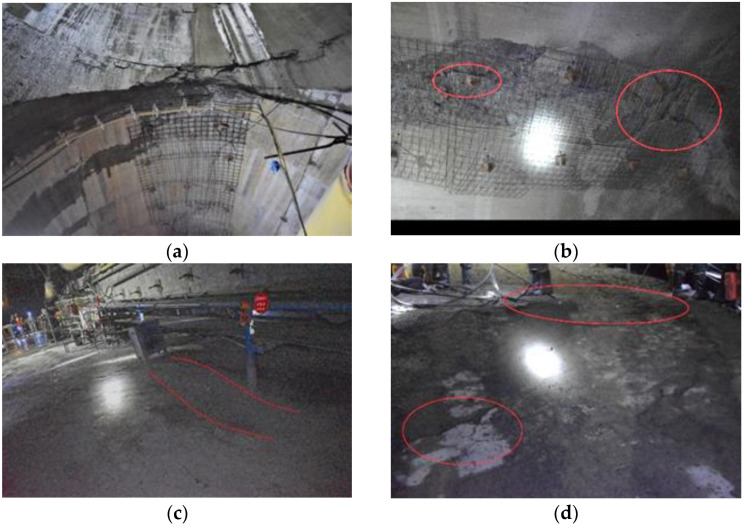
Failure characteristics of the surrounding rock of the horsehead roadway. (**a**) Reinforced concrete lining cracking; (**b**) reinforced concrete lining cracked and falling off; (**c**) floor heave; and (**d**) reinforced concrete floor cracking.

During the construction of the horsehead roadway, there was no obvious failure to the surrounding rock of the two sidewalls. In the middle of the roof, there were cracks in the shotcrete spraying, cracking and falling off of the arch lining, and roof sinking. The roof separation exceeded 45 mm. The floor cracked and heaved, despite multiple floor dinting. The cumulative floor heave was greater than 700 mm, which seriously affected the safety of the underground transportation and construction. The failure characteristics of the roof and floor of the horsehead roadway are shown in Figure 3.

## 3. Influencing Factors of the Deformation and Failure of the Surrounding Rock of the Horsehead Roadway

The cross-section of the horsehead roadway is large, and its stress environment is complex. In order to provide a basis for studying the failure mechanism of the surrounding rock and the optimization of the support scheme design in the return air shaft of the Libi Coal Mine, the main influencing factors of the deformation and failure of the surrounding rock were analyzed as follows using a geological survey, field test, laboratory test, and theoretical analysis.

### 3.1. The Poor Lithology of Argillaceous Surrounding Rock

**Figure 4:**
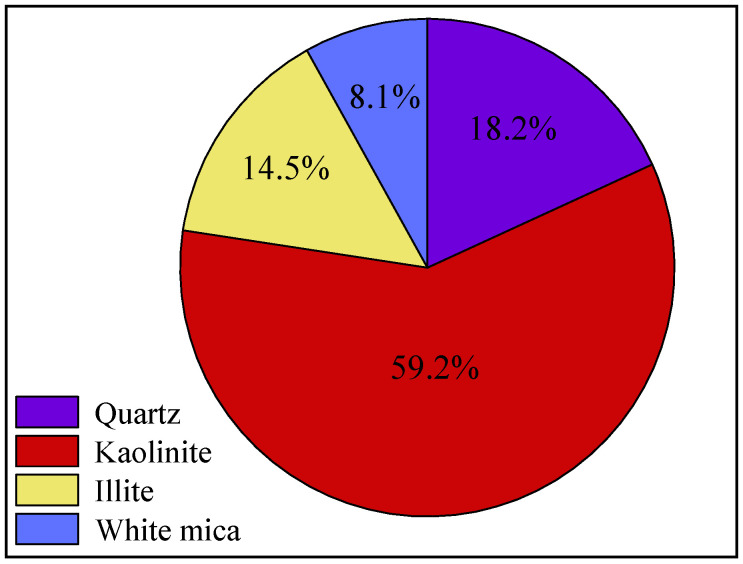
XRD results of the argillaceous surrounding rock.

**Figure 5:**
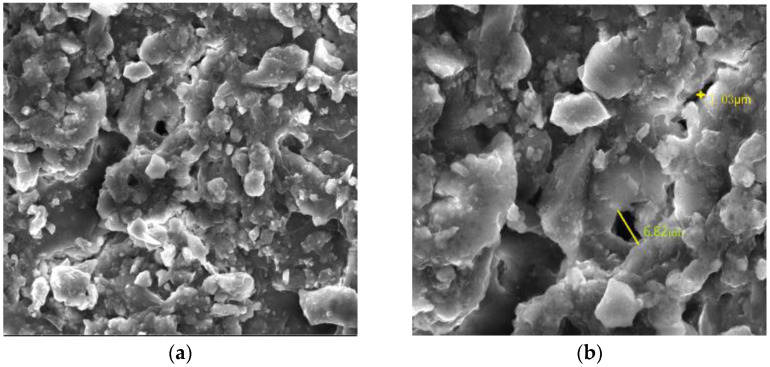
Microstructure of the argillaceous rock: (**a**) amplified 2000 times; and (**b**) amplified 4000 times.

Most of the surrounding rock is mudstone or sandy mudstone with a low strength and poor bearing capacity. Figure 4 shows the X-ray diffraction (XRD) test results of the roof mudstone samples of the horsehead roadway; the contents of the clay minerals kaolinite and illite in the surrounding rock are 59.2% and 14.5%, respectively. Due to the hydrophilicity of clay minerals, when water invades the rock layer, it can easily cause volume expansion and mud disintegration, resulting in a decrease in the overall strength of the surrounding rock. The weak and broken surrounding rock is prone to large deformation and failure. Figure 5 is the scanning electron microscope (SEM) experimental results of the surrounding rock samples. The surface of the rock sample is rough, the cementation is poor, the micropores are more developed, and there is considerable debris on the surface of the rock sample. The maximum width of the fracture is 6.82 μm when the fracture is magnified to 4000 times, and the weak cementation of the rock mass and the existence of the fracture seriously affect the long-term stability of the surrounding rock.

### 3.2. The Small Thickness of Anchorage Layer in the Roof and Insufficient Reinforcement Depth of the Floor

**Figure 6:**
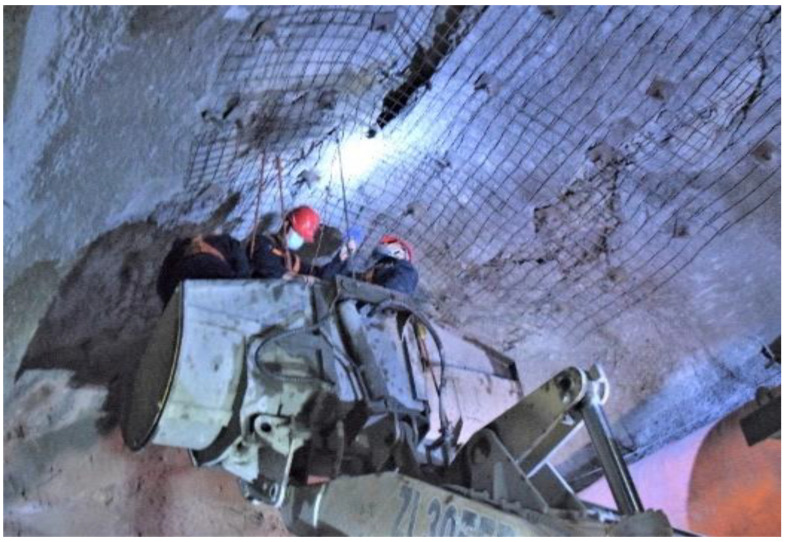
Borehole image monitoring of the surrounding rock of the horsehead roadway.

The ZKXG30 borehole image monitoring camera was used to detect the fracture distribution of the surrounding rock of the roof of the horsehead roadway (Figure 6).

**Figure 7:**
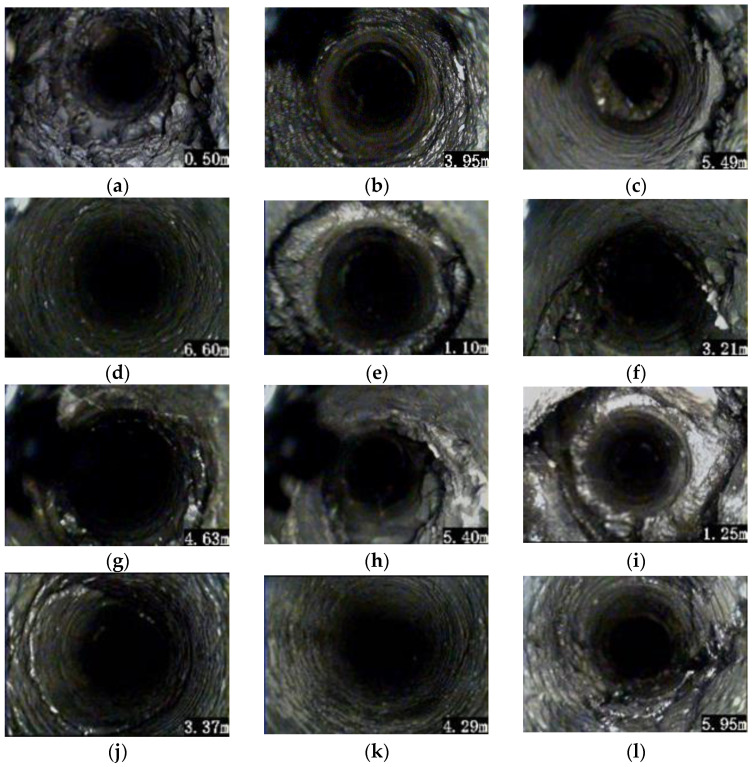
Borehole image of the fractures of the surrounding rock of the horsehead roadway: (**a**) shallow cracks in borehole # 1; (**b**) ring crack in borehole # 1; (**c**) deep fracture in borehole # 1; (**d**) deep rock of borehole # 1 is intact; (**e**) shallow cracks in borehole # 2; (**f**) longitudinal crack in borehole # 2; (**g**) ring crack in borehole # 2; (**h**) deep fracture in borehole # 2; (**i**) shallow cracks in borehole # 3; (**j**) ring crack in borehole # 3; (**k**) the surrounding rock of borehole # 3 is intact; and (**l**) deep fracture in borehole # 3.

**Figure 8:**
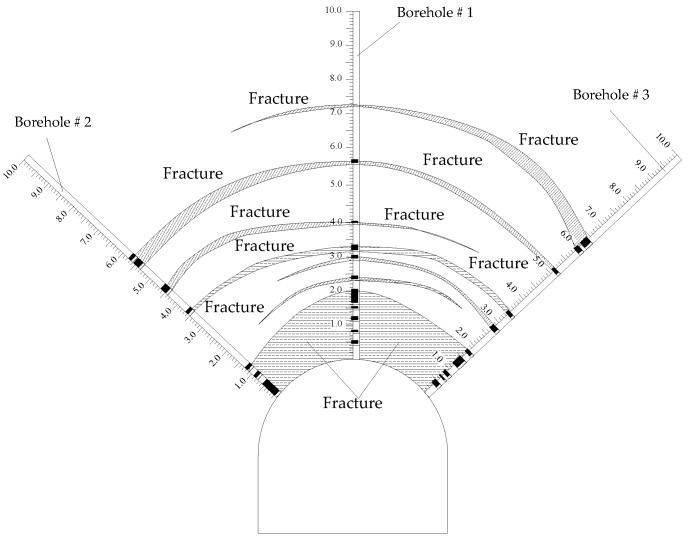
Distribution of the fracture circle of the mudstone roof.

Using the results of the borehole image monitoring of the surrounding rock, the fracture distribution law of the mudstone roof was revealed, as shown in Figure 7 and Figure 8. The fractures in borehole # 1 in the middle of the roof developed to a depth of 0–2.0 m, and the rock was broken. After that, large cracks appeared at the depths of 3.95 m and 5.5 m, and the rest was relatively complete. Borehole # 2 in the left shoulder of the roof showed a fracture zone with a depth of 0–1.1 m with considerable fracture development; larger fractures exist at 3.5 m, 4.5 m, and 5.3–5.7 m, and the others were relatively complete. The rock mass of borehole # 3 in the right shoulder of the roof were broken with a depth of 0–1.5 m. After that, there were fractures at 2.8 m and 3.5 m. There was a fracture at a depth of about 6.0 m. The rock with a depth exceeding 6.0 m was relatively complete with few cracks.

The failure depth of the roof is greater than the anchorage depth of the bolt (2.5 m), which makes the anchorage fail with ease, and the active support of the bolt on the surrounding rock does not work. The borehole image monitoring shows that the separation occurs outside of the anchorage zone of the bolt, so the insufficient thickness of the roof anchorage layer is an important factor for roof failure.

The floor of the horsehead roadway is reinforced with 400 mm thick flat-bottomed reinforced concrete. The reinforcement depth is in an inefficient reinforcement area. Due to the superposition of high in situ stress and additional stress from the shaft, the floor, with a low support strength, becomes the main place where the pressure relief of the surrounding rock takes place. The failure of the floor leads to the progressive damage and deformation failure of the surrounding rock of the roadway, which is not conducive to the long-term stability of the surrounding rock.

### 3.3. The Superimposed Influence of Additional Stress from the Shaft and Construction Disturbance

The horsehead roadway is in a high-stress state. The additional stress of the shaft and the influence of the subsequent roadway construction led to stress superposition in the horsehead roadway, high-stress concentration, and complex stress state, which adversely affect the stability of the surrounding rock of the horsehead roadway.

### 3.4. Horizontal Tectonic Stress

The greater the angle between the direction of the maximum principal stress and the direction of the roadway, the worse the stability of the roof and floor, and the more severe the mine pressure [18]. According to the in situ stress test and analysis of the Jincheng mining area [19] and the Libi Coal Mine, the maximum horizontal principal stress of the Libi Coal Mine is 16.1 MPa–17.2 MPa, with an average of 16.5 MPa; the minimum horizontal principal stress is 8.4 MPa–9.8 MPa, with an average of 8.9 MPa. The vertical stress is 12.5 MPa–13.1 MPa, with an average of 12.9 MPa. The maximum horizontal principal stress direction is N82° E, which is nearly east–west. The axis of the horsehead roadway of the return air shaft in the Libi Coal Mine is in the north–south direction (NW11°), and the axis direction of the horsehead roadway is approximately perpendicular to the direction of the maximum horizontal principal stress (intersection angle of 87°), which is not conducive to the long-term stability of the surrounding rock of the horsehead roadway, especially the roof and floor.

## 4. Deformation and Failure Mechanism of the Surrounding Rock of the Horsehead Roadway

### 4.1. Numerical Model and Simulation Scheme

(1) Modelling

**Figure 9:**
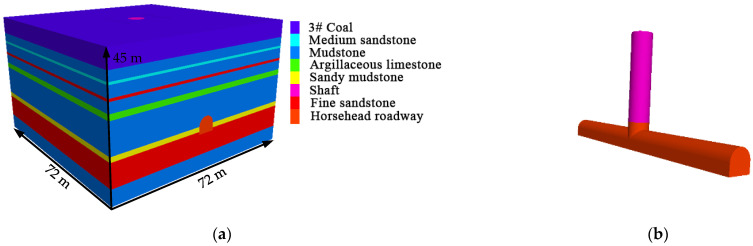
Numerical model: (**a**) numerical model diagram and (**b**) the horsehead roadway.

The FLAC3D numerical model was established, as shown in Figure 9, according to the actual engineering geological conditions of the horsehead roadway of the return air shaft in the Libi Coal Mine. The section size of the horsehead roadway is 6.5 × 5.95 m (width × height), and the model size is 72 m × 72 m × 45 m (length × width × height). A vertical stress of 12.85 MPa was applied to the upper boundary of the model, which represents the pressure of the overlying strata, with horizontal displacement fixed at the lateral boundary and both horizontal and vertical displacements fixed at the bottom boundary. The Mohr–Coulomb yield criterion was adopted. The entire model and the horsehead roadway model are shown in Figure 9, and the mechanical parameters of the rock mass are shown in Table 1.

(2) Simulation program

According to the occurrence conditions and surrounding rock failure characteristics of the horsehead roadway of the air return shaft in the Libi Coal Mine, the two main influencing factors—additional stress and horizontal tectonic stress—were simulated and analyzed. The simulation scheme is as follows:

(a) The fixed lateral pressure coefficient of the in situ stress was 1.0, and the 2 cases of shaft and non-shaft were simulated to obtain the law of influence of additional stress in the shaft on the stability of the surrounding rock of the horsehead roadway, including stress distribution, plastic zone distribution, and deformation law.
(b) The lateral pressure coefficients of 1.0, 1.2, 1.4, 1.6, 1.8, and 2.0 were simulated to analyze the influence law of the different horizontal tectonic stresses on the stability of the surrounding rock of the horsehead roadway, including stress distribution, plastic zone distribution, and deformation law.

### 4.2. Influence of the Additional Stress of the Shaft on the Stability of the Surrounding Rock

**Figure 10:**
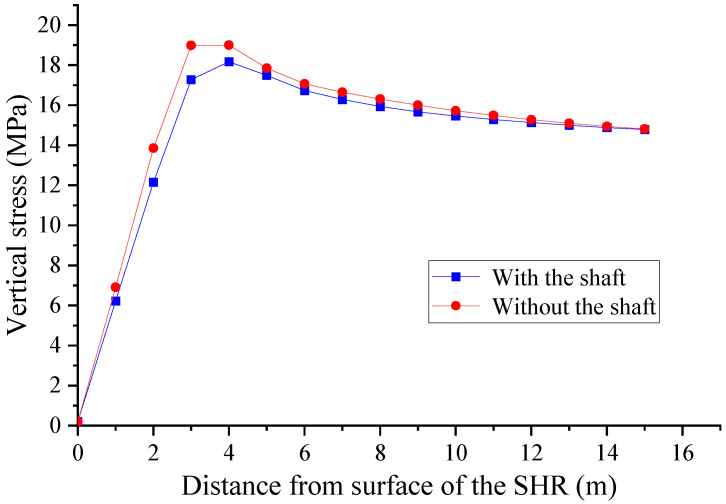
Vertical stress distribution of the surrounding rock of the horsehead roadway (SHR: sidewall of the horsehead roadway).

**Figure 11:**
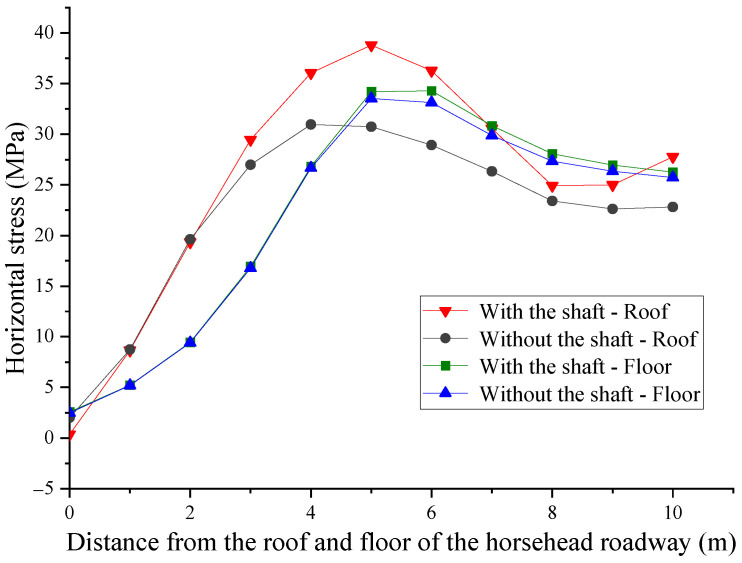
Horizontal stress distribution of the surrounding rock of the horsehead roadway.

A vertical stress monitoring measuring line along the horizontal direction was set at the sidewalls center of the horsehead roadway at a distance of 5 m from the shaft. A horizontal stress monitoring measuring line in the vertical direction was set at the center of the roof and floor of the horsehead roadway at a distance of 5 m from the shaft. The vertical and horizontal stress distribution, respectively, of the surrounding rock of the horsehead roadway with and without the shaft are shown in Figure 10 and Figure 11.

The maximum vertical stress of the surrounding rock without the shaft is 19.4 MPa, which is 3 m away from the surface of the roadway sidewall. When the shaft is present, the maximum vertical stress of the surrounding rock is 18.4 MPa, which is 4 m away from the surface of the sidewall of the horsehead roadway. The presence of the shaft has little effect on the vertical stress distribution of the surrounding rock of the horsehead roadway.

After the excavation of the horsehead roadway, the horizontal stress of the roof and floor first increases and then decreases, finally approaching the in situ stress. The presence of the shaft has a great influence on the horizontal stress of the roof of the roadway. The maximum horizontal stress of the roof is 34.1 MPa when there is no shaft, whilst the maximum horizontal stress of the roof is 39.2 MPa when there is a shaft. The presence of the shaft makes the roof stress concentration more obvious and increases the distribution range of the high-stress areas, which can cause roof damage with ease. The difference between the horizontal stress variation curves of the floor in the two cases is not significant, indicating that the shaft has little effect on the horizontal stress distribution of the floor.

**Figure 12:**
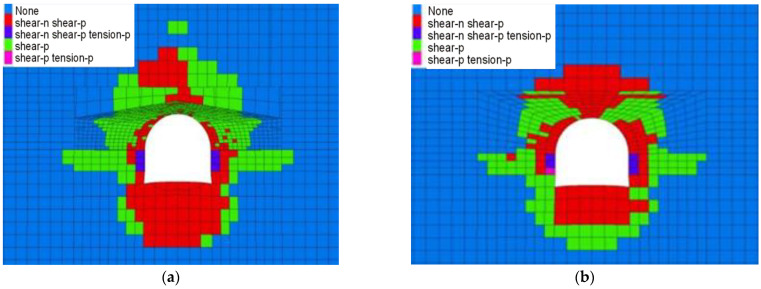
Distribution law of the plastic zone of the surrounding rock: (**a**) with a shaft and (**b**) without s shaft.

In the absence of a shaft, the depths of the shear failure zone of the roof and floor are 4 m and 3 m, respectively (Figure 12). In the presence of the shaft, the depth of the shear failure zone of the roof and floor is extended to 5 m, and the additional stress of the shaft leads to an increase in the plastic zone of the surrounding rock. The progressive damage and deformation failure of the surrounding rock is caused by being exposed to high stress over time. Therefore, in order to ensure the long-term stability of the surrounding rock, it is necessary to increase the thickness of the anchorage ring in the horsehead roadway near the shaft to support the cross-border.

As shown in Table 2, compared to the deformation of the surrounding rock without the shaft, the roof subsidence increased by 8.4%, the floor heave increased by 9.3% and the side deformation increased by 21.8% in the condition with a shaft. When the shaft exists, the deformation range of the roof and floor is large, which can cause roof separation and floor heave easily. Due to the influence of additional stress from the shaft, the deformation of the surrounding rock in the presence of the shaft is greater than that without the shaft.

### 4.3. Influence of horizontal Tectonic Stress on the Stability of the Surrounding Rock

**Figure 13:**
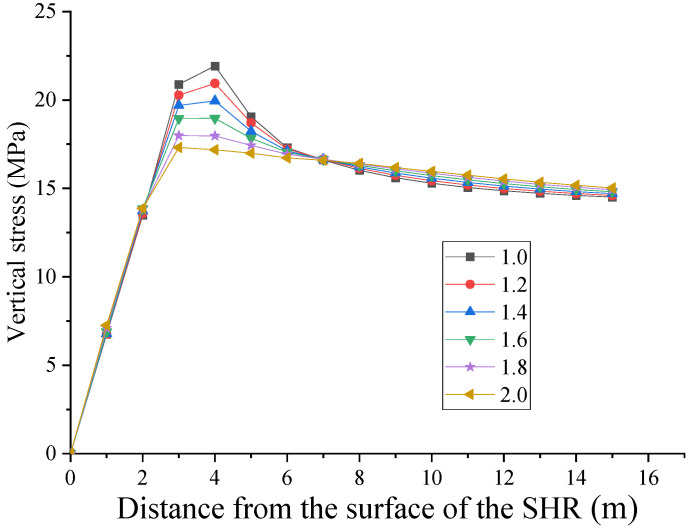
Vertical stress distribution of the surrounding rock under different lateral pressure coefficients (SHR: side of the horsehead roadway).

**Figure 14:**
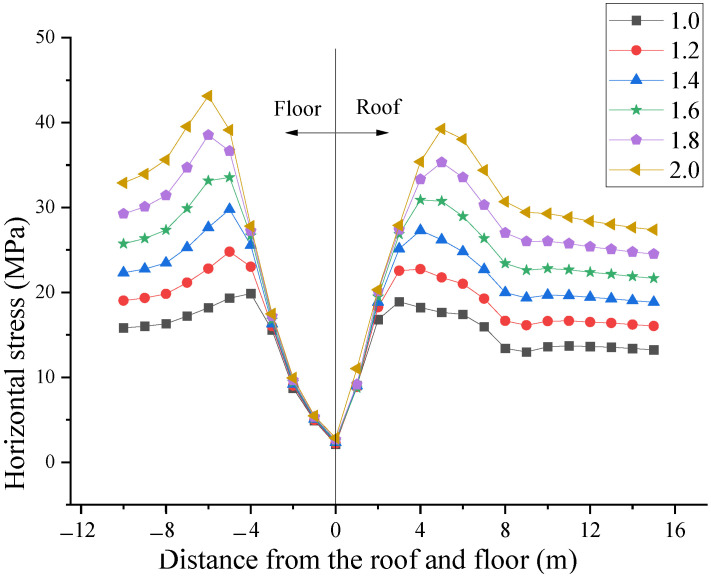
The horizontal stress distribution law of the surrounding rock under different lateral pressure coefficients.

Under the influence of different lateral pressure coefficients (horizontal tectonic stress), the vertical stress and horizontal stress distribution of the surrounding rock of the horsehead roadway at 5 m from the shaft are shown in Figure 13 and Figure 14, respectively.

As the lateral pressure coefficient increases, the vertical stress peak of the surrounding rock gradually decreases, and the stress peak gradually approaches the roadway surface (Figure 13). The peak vertical stress reaches its maximum at 21.9 MPa with a lateral pressure coefficient of 1.0, at 4 m from the roadway surface. The peak vertical stress reaches its minimum at 17.3 MPa with a lateral pressure coefficient of 2.0, at 3 m from the roadway surface.

As the lateral pressure coefficient increases, the peak horizontal stresses of the roof and floor also gradually increase, and the position of the peak stresses gradually move away from the roadway (Figure 14). In the process of increasing the lateral pressure coefficient from 1.0 to 2.0, the peak stress of the roof increases from 18.9 MPa to 39.2 MPa, and the position of peak stress increases from 3 m to 5 m from the horsehead roadway. The peak stress of the floor increases from 19.9 MPa to 39.5 MPa, and the position of peak stress increases from 4 m to 7 m from the horsehead roadway floor.

**Figure 15:**
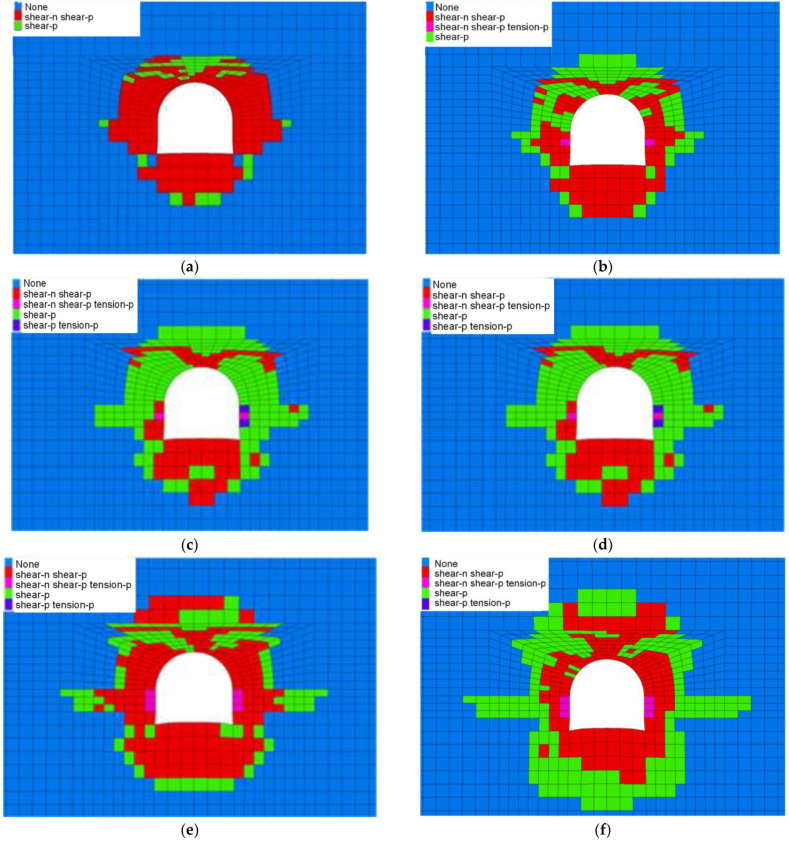
Distribution law of the plastic zone of the surrounding rock under different lateral pressure coefficients. (**a**) 1.0. (**b**) 1.2. (**c**) 1.4. (**d**) 1.6. (**e**) 1.8. (**f**) 2.0.

As shown in Figure 15, as the lateral pressure coefficient increases, the plastic zone range of the horsehead roadway roof increases from 3 m to 6 m, and the plastic zone range of the floor increases from 4 m to 6 m. The failure of the surrounding rock is mainly shear failure. When the lateral pressure coefficient is greater than 1.4, the zone of the tensile shear failure appears in the shallow surrounding rock of the two sides of the horsehead roadway.

**Figure 16:**
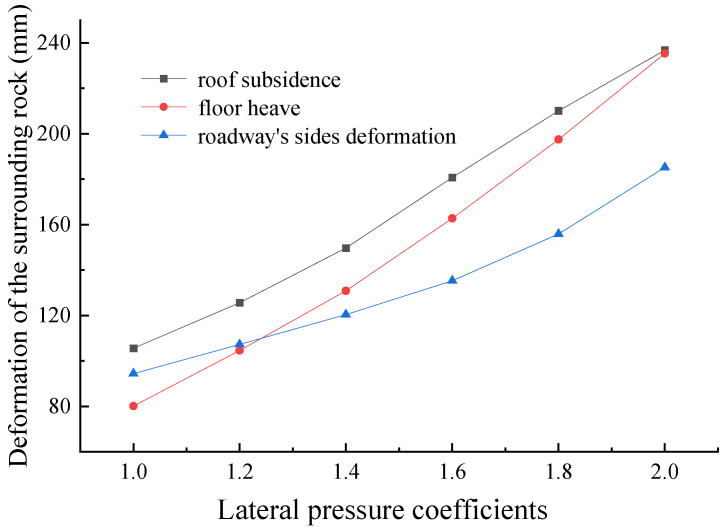
Displacement of the surrounding rock under different lateral pressure coefficients.

As shown in Figure 16, as the lateral pressure coefficient increases, the deformation of the surrounding rock gradually increases. In the process of increasing the lateral pressure coefficient from 1.0 to 2.0, the roof subsidence increases from 105.6 mm to 236.8 mm, the floor heave increases from 80.1 mm to 235.4 mm, and the deformation increases significantly.

## 5. Control Principles and Countermeasures for the Argillaceous Surrounding Rock of the Horsehead Roadway

### 5.1. The Principle of Controlling the Surrounding Rock of the Horsehead Roadway

According to the failure characteristics of the roof and floor of the horsehead roadway, combined with the distribution law of the fracture circle of the mudstone roof and the failure mechanism of the horsehead roadway, the basic principles for the control of the stability of the surrounding rock of the horsehead roadway are proposed in what follows:

(1) The thickness of the anchorage layer should be increased. The drilling detection results of the muddy surrounding rock of the horsehead roadway’s roof show that the surrounding rocks are broken within 3.5 m of the roof, and the separation layer appears outside the bolt anchorage zone. The muddy surrounding rocks of the bolt drilling can be broken easily and lead to anchorage failure. It is difficult to create an effective support for the roof using a conventional bolt support or increasing the support density. Therefore, the thickness of the anchorage layer should be increased, and the strength and integrity of the surrounding rock should be improved using prestressed full-length anchorage technology.
(2) The floor reinforcement breaks through the minimum reinforcement depth and forms a closed structure with the roof and sidewalls. One of the main reasons for the failure of the floor heave is the insufficient reinforcement depth for the floor. In order to form an effective bearing structure on the floor, the reinforcement depth of the floor should exceed the minimum reinforcement depth. Moreover, the floor heave has long-term rheological properties due to the high stress of the horsehead roadway. In order to ensure the long-term stability of the floor, a closed support structure should be formed to prevent the crack from expanding deeply; thus, the floor and the two sidewalls can form a whole to enhance the overall stability of the surrounding rock.
(3) Some key positions should be reinforced. Some corners and other locations with stress concentration should be properly strengthened so that the surrounding rock forms a stable weight-bearing structure to maintain its long-term stability.

### 5.2. Prestressed Full-Length Anchorage Technology for the Mudstone Roof

The prestressed full-length anchoring technology has the characteristics of high pre-load, rapid resistance increasing, and high resistance. The mudstone roof was reinforced with a prestressed full-length anchoring support [20], which has the following advantages: (1) Good structural stability: the full-length anchoring bolt or anchor cable orifices are less stressed, avoiding orifice damage and maintaining efficient support. (2) High support sensitivity: sensitive to the deformation of the surrounding rock, rapid increase in resistance, and timely containment of the deformation. (3) Strong shear capacity: when the interface is staggered, the rod rock plays a shear role immediately. (4) High anchorage stiffness: compared with the free section of the end anchor, which is detached from the rock, the entire length of the anchor rope interacts with the rock, and the anchorage stiffness increases considerably. (5) Anticorrosion: the inherent strength of the anchor ropes is maintained, and the service life of the anchor ropes increases.

**Figure 17:**
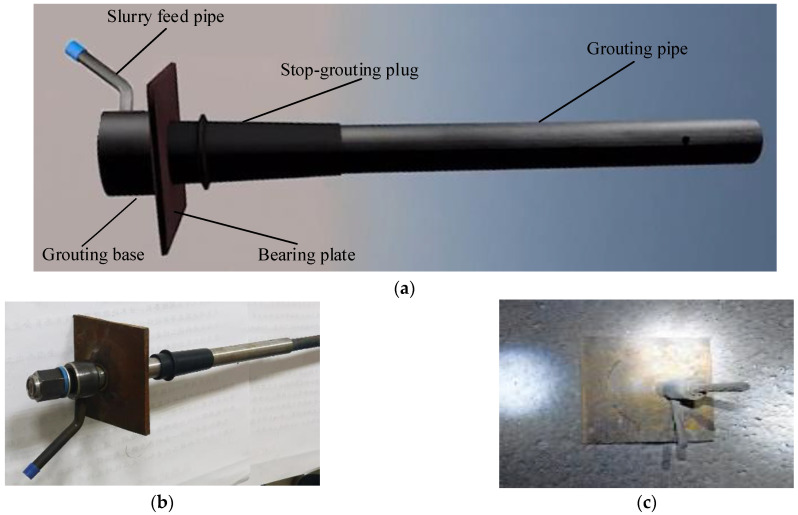
Innovative anchor-grouting device for prestressed full-length anchorage: (**a**) structure of anchor-grouting device; (**b**) the innovative anchor-grouting device; and (**c**) field application.

Combined with the borehole image monitoring results of the fracture distribution of the surrounding rock in the horsehead roadway, we propose the prestressed full-length anchorage technology of bolts or cables based on an innovative anchor-grouting device because the depth of the roof failure is greater than the depth of the bolt anchorage, so the thickness of the anchorage ring has to be increased and the strength and integrity of the rock mass have to be improved. The anchor-grouting device (Figure 17) was used with cables. As shown in Figure 17a, the anchor-grouting device mainly includes the grouting base, slurry feed pipe, stop-grouting plug, bearing plate, and grouting pipe. The pre-tightening force of the cables was first tensioned after anchoring using a resin agent, and then the full-length anchorage was completed with grouting through the grouting device. Compared with the traditional hollow grouting cable, there is no strength loss in the cable body for the prestressed full-length anchorage support technology. The combination of anchor-grouting devices with cable support plays three roles—pre-tightening, full-anchorage, and grouting, which can effectively construct the thick prestressed bearing ring and achieve the long-term stability of the surrounding rock.

In addition, as an anchor cable support that is too long does not produce a significant effect on the control of the surrounding rock, the anchor cable can be shortened appropriately. According to the drilling detection results of the fracture distribution of the surrounding rock of the roof, there is almost no fracture development in the roof beyond 6.0 m in depth. The original support scheme with an anchor cable of 8.3 m was too long, resulting in a waste of support materials. The length of the anchor cable was shortened from 8.3 m of the original support during the excavation.

### 5.3. Active and Passive Reinforcement Technology of the Anchor Cable and Inverted Arch for the Floor

The floor anchor cable can reduce the sliding between the structural planes of the floor rock mass and enhance the stiffness and strength of the floor. The inverted arch can make the surrounding rock of the floor form a closed structure, resist the stress extrusion of the surrounding rock mass to its greatest extent, and improve the supporting effect. The active and passive reinforcement technology of the floor anchor cable and inverted arch can form a closed structure in the floor while enhancing the strength of the floor. The deep stable rock layer of the floor was used to control the large deformation of the shallow surrounding rock of the floor and maintain long-term stability. In addition, the bottom corners is in the stress concentration area, and the bolt in the bottom corners is arranged at the bottom corners of the two sidewalls to enhance the strength of the surrounding rock of the side angle and the bottom corners.

When reinforcing the surrounding rock of the roadway, there is a reasonable reinforcement range, so that the support body and the surrounding rock form an effective reinforcement area. According to the theory of effective reinforcement depth, the minimum reinforcement depth of the floor was calculated by the following formula [21]:

(1) $$H={\;H}_{1\;}+H_{2}+∆H$$

where H_1_ is the depth of the invalid reinforcement area; H_2_ is the minimum thickness of the bearing ring formed by the support body; and ∆H is the surplus coefficient.

H_1_ can be derived from Equation (2):

(2) $$H_{1}=2R\;-\;h=2\;\times \;3.25\;-\;5.95=0.55\;m$$

where R is the equivalent radius of the roadway, where half of the width of the horsehead roadway is taken, that is, 3.25 m; and h is the height of the horsehead door, considered as 5.95 m.

Generally, when the buried depth of the roadway is 400 m, the minimum bearing ring thickness is 2.0 m; when the buried depth is 600 m, the minimum bearing ring thickness is 2.5 m; and the minimum bearing ring thickness is 3.0 m when the buried depth is 800 m. The buried depth of the horsehead roadway in the Libi Coal Mine is 537 m, and the minimum bearing ring thickness is 2.5 m.

The surplus coefficient “∆H” usually considers the influence of lithology, service time, and anchorage quality, and is 0.2–0.8 m. According to the condition of the horsehead roadway of the return air shaft in the Libi Coal Mine, 0.5 m was used.

In summary, the minimum reinforcement depth of the floor should be greater than 3.6 m.

$$H=H_{1}+H_{2}+∆H=0.55+2.5+0.5=3.6\;m$$

Considering the exposed length of the floor anchor cable and the influence of the inverted arch, the length of the floor anchor cable was 4.3 m. By comparing various experiences and considering the difficulty of construction, the height of the inverted arch was 0.8 m.

### 5.4. Numerical Simulation of the Reinforcement Scheme of the Surrounding Rock of the Horsehead Roadway

FLAC3D 5.0 software was used to numerically analyze the reinforcement scheme of the roof and floor. The model and mechanical parameters are shown in Figure 10 and Table 1. The depth of the horsehead roadway was 537 m. The specific simulation scheme is as follows.

(1) Scheme 1: The roof was reinforced with five anchor cables, each 21.8 mm in diameter and 7200 mm in length based on the original support scheme (thirteen thread steel bolts in the roof and three on both sidewalls, each 22 mm in diameter and 2500 mm in length). The interval and spacing of bolts were both 1600 mm.
(2) Based on scheme 1, active and passive reinforcement techniques, including the prestressed anchor cable and the reverse arch, were adopted for the floor. Five anchor cables were used for the floor, each 21.8 mm in diameter and 4300 mm in length. The pre-tightening force was 180 kN. The interval and spacing of bolts were 1100 mm and 1000 mm, respectively. One anchor bolt, each 22 mm in diameter and 2800 mm in length, was constructed at the bottom corners of both sidewalls. The reverse arch was 0.8 m high.

**Figure 18:**
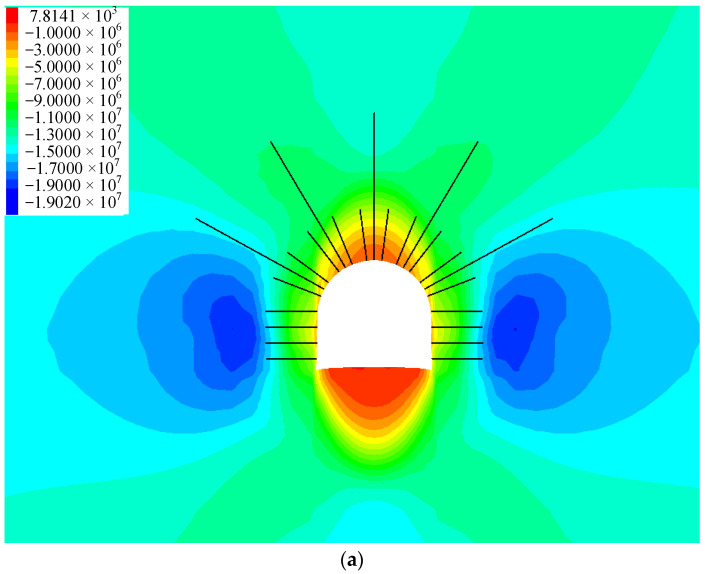

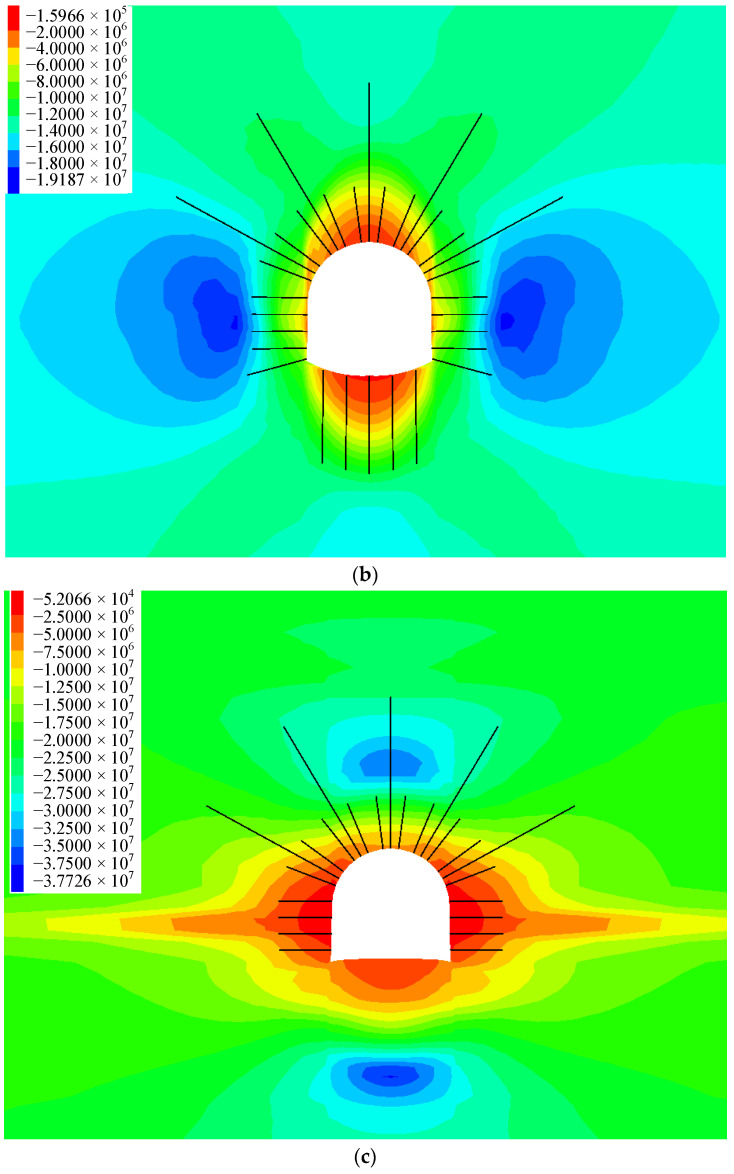

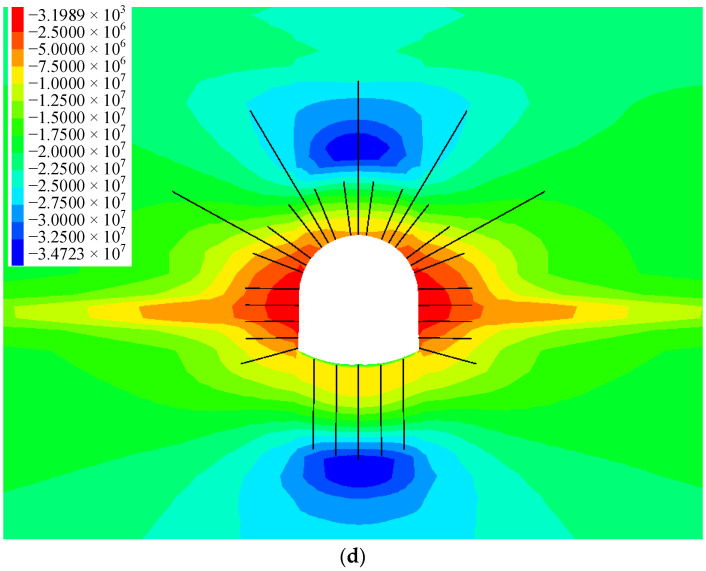
Stress distribution of the different supporting schemes at 5 m from the shaft (unit: Pa): (**a**) vertical stress for scheme 1; (**b**) vertical stress for scheme 2; (**c**) horizontal stress for scheme 1; and (**d**) horizontal stress for scheme 2.

The nephograms of the vertical and horizontal stress distribution of the surrounding rock at 5 m from the shaft after using two different support schemes are shown in Figure 18.

From Figure 18, it can be seen that the peak vertical stress values of the surrounding rock in schemes 1 and 2 are 19 MPa and 19.2 MPa, respectively, with little difference. However, after supporting the floor, the tensile stress area of the floor disappears. As shown in Figure 18b, after the reinforcement of the floor, all the areas below the inverted arch become compressive stress areas, and the failure capacity of the floor is greatly enhanced. When the two different schemes are supported, the horizontal stress of the roof is not much different, but the horizontal stress of the floor is obviously different. The peak values of the horizontal stress of the floor are 37.7 MPa and 34.7 MPa, respectively, for the two schemes. Therefore, increasing the bottom corners bolt and the floor anchor cable not only improves the strength of the floor but also improves the stress environment of the floor and reduces the stress concentration of the floor, which is conducive to the long-term stability of the floor.

The displacement of the roof and floor of the horsehead roadway at 5 m from the shaft under the two different reinforcement schemes and the displacement of the roof and floor under the original support scheme are shown in Table 3.

From Table 3, it can be seen that, after the roof is supported by the full-length anchoring technology (scheme 1), the deformation of the surrounding rock of the roof is reduced from 193.5 mm of the original support scheme to 151.6 mm, a reduction of 21.7% compared with the original scheme, and the floor heave is not much different from the original scheme. Based on scheme 1, the amount of floor heave is reduced from 177.7 mm to 78.2 mm after using the active and passive reinforcement technology of anchor cable and inverse arch, with a decrease of 60.0%. In summary, the prestressed full-length anchoring technology of the roof and the anchor cable and inverse arch active and passive reinforcement technology of the floor can effectively reduce the deformation of the surrounding rock of the horsehead roadway.

## 6. Reinforcement Schemes for the Argillaceous Surrounding Rock of the Horsehead Roadway and Field Test

### 6.1. Reinforcement Schemes for the Horsehead Roadway

**Figure 19:**
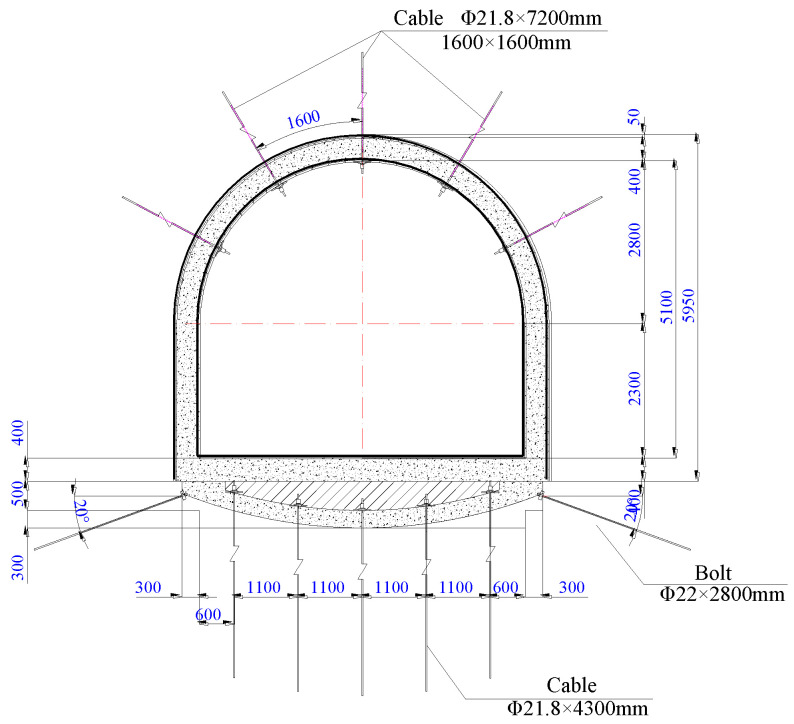
Reinforcement schemes for the horsehead roadway.

In order to control the large deformation of the surrounding rock of the horsehead roadway, the roof was reinforced using the prestressed full-length anchoring technique with the innovative anchor-grouting device, and, for the floor, the active and passive reinforcement technology of cables and inverse arch were adopted. The reinforcement scheme is shown in Figure 19.

The roof was fitted with 5 cables, each 21.8 mm in diameter and 7200 mm in length. Each cable was equipped with a set of anchoring devices. The interval and spacing of cables were both 1600 mm. Two MSZ2360 resin agents and one MSK2335 resin agent were used to anchor each cable. The pre-tightening force was not less than 180 kN. The full-length anchorage was achieved by grouting with the anchoring device.

One HRB500 steel bolt was attached to the bottom corner of both sidewalls, with a diameter of 22 mm and a length of 2800 mm and a row spacing of 800 mm. The inverse arch of the floor was 0.8 m high. The main reinforcement of the inverse arch was an HRB400 steel bar with a diameter of Φ 20 mm. The longitudinal and transverse spacing between the main reinforcements was 200 mm. After the construction of the inverse arch was completed, the concrete was backfilled to the flat floor, and then the floor was hardened. The concrete strength was C30.

### 6.2. Field Monitoring and Maintenance Effect

**Figure 20:**
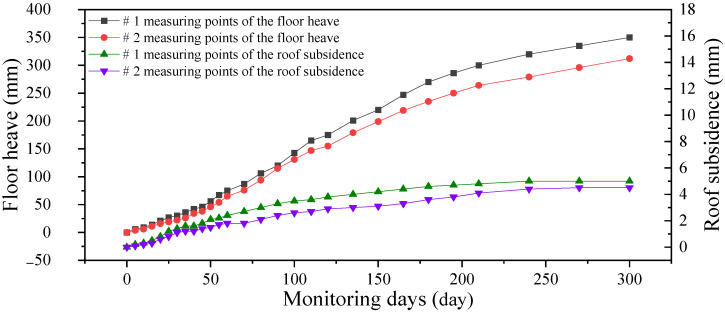
Displacement of the roof and floor of the horsehead roadway.

The prestressed cables and anchor-grouting device to reinforce the roof of the horsehead roadway were implemented, and the floor was excavated several times due to the floor heave. Because the Libi Coal Mine is in the construction period, the main roadway of the shaft bottom has not yet been connected, and the construction schedule is tight. The floor of the horsehead roadway has not yet been fitted with an inverse arch and floor anchor cable. The measuring points # 1 and # 2 were arranged at 5 m and 8.5 m away from the shaft, respectively, and the field observation was conducted from 14 November 2020 to 11 September 2021. The deformation of the surrounding rock is shown in Figure 20.

**Figure 21:**
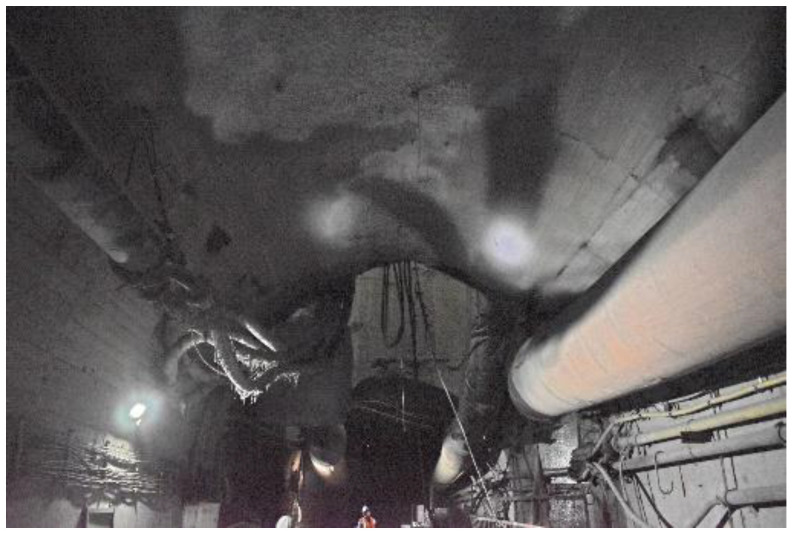
Maintenance of the horsehead roadway.

After the new prestressed full-length anchoring technology was adopted for the mudstone roof, the roof subsidence was only about 5 mm, showing the remarkable control effects of the new prestressed full-length anchoring technology. The reinforcement effect of the horsehead roadway is shown in Figure 21.

After being undercover several times, the amount of floor heave increased slowly. Considering the creep and rheological properties of the surrounding rock, after the bottom yard and main roadway are connected, the floor anchor cable and inverse arch active and passive reinforcement technology should be adopted to ensure the long-term stability of the surrounding rock of the floor of the horsehead roadway.

## 7. Conclusions and Suggestions

(1) According to the engineering and geological conditions of the horsehead roadway in the Libi Coal Mine, the physical properties of the surrounding rock, and the borehole image monitoring results of the fracture distribution of the surrounding rock, it is concluded that the main factors that affect the deformation and failure of the surrounding rock include the poor lithology of the argillaceous rock surrounding the area, the horizontal tectonic stress, the superimposed influence of additional stress from the shaft and construction disturbance, the small thickness of the roof anchorage layer, and the insufficient reinforcement depth of the floor.
(2) Compared with the absence of a shaft, the presence of a shaft has a greater impact on the horizontal stress of the roof, which increases the peak value of the horizontal stress of the roof and the range of stress concentration. The additional stress of the shaft increases the plastic zone of the surrounding rock. The progressive damage and deformation failure of the surrounding rock under high stress is caused over time. The change in horizontal tectonic stress has a great influence on the stability of the surrounding rock. With the increase in tectonic stress, the stress concentration of the surrounding rock is more evident, and the range of the plastic zone and deformation of the surrounding rock significantly increase.
(3) According to the failure characteristics of the surrounding rock of the horsehead roadway of the return air shaft in the Libi Coal Mine, the law of fracture distribution of the mudstone roof, and the failure mechanism of the surrounding rock, the control principles of the stability of the surrounding rock of the horsehead roadway were presented, including increasing the thickness of the anchorage layer, exceeding the minimum reinforcement depth of the floor, and strengthening the support for the key parts. The prestressed full-length anchorage technology based on the innovative anchor-grouting device for the mudstone roof and active and passive reinforcement techniques of the cables and the inverse arch for the floor was proposed. Additionally, the corner bolts were added to strengthen the support of the key parts.
(4) This new prestressed full-length anchoring technology was used to reinforce the mudstone roof. The field measurement results show that the control effect of the new prestressed full-length anchorage was remarkable, and the roof subsidence was about 5 mm. Floor heave increased slowly after floor dinting. Considering the creep and rheological properties of the surrounding rock, the floor should be reinforced after the bottom pit and the main roadways are connected. The minimum reinforcement depth of the floor should exceed 3.6 m to ensure the long-term stability of the surrounding rock of the horsehead roadway.

## Figures and Tables

**Table 1:** Mechanical parameters of the roof and floor footwall and roadway wall.

Lithology	Thickness (m)	Density (kg/m^3^)	Bulk Modulus (GPa)	Shear Modulus (GPa)	Angle of Internal Friction (°)	Cohesion (MPa)	Tensile Strength (MPa)
Coal 3#	6.0	1430	2.12	1.10	29	0.8	0.50
Mudstone	3.1	2389	1.22	0.60	32	0.9	2.09
Medium sandstone	0.8	2598	5.87	3.97	40	1.1	4.77
Mudstone	3.6	2389	1.22	0.60	32	0.9	2.09
Fine sandstone	3.3	2389	1.32	0.70	32	1.0	2.81
Mudstone	1.9	2389	1.22	0.60	32	0.9	2.09
Argillaceous limestone	5.2	2232	2.42	1.76	29	1.35	2.60
Mudstone	5.0	2389	2.01	1.98	35.1	2.9	3.41
Sandy mudstone	1.7	2238	2.04	1.65	33	0.83	0.55
Fine sandstone	8.0	2389	1.32	0.70	32	1.0	2.81
Mudstone	5.4	2389	1.22	0.60	32	0.9	2.09

**Table 2:** Displacement of the surrounding rock 5 m away from the shaft, in conditions with and without a shaft.

	Deformation (mm)	
	with a Shaft	without a Shaft
Roof subsidence	193.5	178.5
Floor heave	177.7	162.6
Sidewall deformation	164.6	135.1

**Table 3:** Vertical displacement of the roof and floor under the different support schemes (unit: mm).

	Deformation			Scheme 1 Change Rate	Scheme 2 Change Rate
	Original Scheme	Scheme 1	Scheme 2		
Roof subsidence	193.5	151.6	143.6	21.7%	25.8%
Floor heave	177.7	175.2	78.2	1.4%	60.0%

## Data Availability

All the data analyzed during this study are included in the published article.
